# Expression of MMP-2, −7, −9, MT1-MMP and TIMP-1 and −2 has no prognostic relevance in patients with advanced epithelial ovarian cancer

**DOI:** 10.3892/or.2011.1608

**Published:** 2012-12-22

**Authors:** JEAN-LUC BRUN, ANNIE CORTEZ, BÉNÉDICTE LESIEUR, SERGE UZAN, ROMAN ROUZIER, EMILE DARAÏ

**Affiliations:** 1 Department of Gynecology, Hôpital Tenon, AP-HP, F-75571 Paris; 2 Department of Pathology, Hôpital Tenon, AP-HP, F-75571 Paris; 3 Department of Obstetrics and Gynecology, Hôpital Pellegrin, Université Bordeaux Segalen, F-33076 Bordeaux; 4 UPMC University Paris 06, UPRES EA 4053, F-75005 Paris, France

**Keywords:** matrix metalloproteinase, prognosis, ovarian neoplasms

## Abstract

Matrix metalloproteinases (MMPs) and their tissue inhibitors (TIMPs) are involved in tumor invasion, but their prognostic significance is still under discussion. We set out to analyze the epithelial and stromal expression of MMP-2, MMP-7, MMP-9, MT1-MMP, TIMP-1 and TIMP-2 in advanced epithelial ovarian cancers and to assess their prognostic value. A tissue microarray of malignant ovarian tumors from 69 patients was constructed. Immunostaining results were scored using the HSCORE and assessed by univariate analysis with Bonferroni correction and classical multidimensional scaling (CMDS). Kaplan-Meier survival curves calculated with regard to patient and tumor characteristics were compared by the log-rank test. Patients treated by primary surgery (n=43) had a higher tumor size and a trend toward higher epithelial MMP and TIMP expression than those treated by interval surgery (n=26). Optimal cytoreduction (residue ≤1 cm) was obtained in 27 and 18 patients, respectively. Clinical and histological characteristics were not different in patients with optimal cytoreduction and those with suboptimal cytoreduction. The expression of epithelial MMP-9 (P=0.002) and TIMP-2 (P=0.026) were higher in the latter group. CMDS failed to demonstrate any influence of MMP and TIMP expression with regard to cytoreduction outcome. MMP and TIMP expression did not influence survival. Their prognostic values were outweighed by histological type, lymph node involvement and cytoreduction. Standard statistical analysis adjusted after Bonferroni correction and CMDS reduced the relevance of MMPs and TIMPs in the prognosis of patients with advanced ovarian cancer.

## Introduction

Matrix metalloproteinases (MMPs) and their tissue inhibitors (TIMPs) are controllers of extracellular matrix turnover. Both MMP and TIMP expressions are found to be altered in benign and malignant tumors, as well as during invasion and metastasis which require the breakdown and removal of extracellular matrix (1).

The presence of MMPs in ovarian cancer and their contribution to the invasive phenotype of these tumors have been documented in both *in vitro* and clinical studies. Immunochemistry and Western blot analysis have shown higher activated MMP-2 in epithelial ovarian carcinomas than in benign tumors (2,3). High levels of MMP-9 have been found in human ovarian carcinoma xenografts and overexpression of MMP-7 in ovarian cancer cell lines and cancer surgical specimens (3,4). MT1-MMP has been shown to regulate cell proliferation, cell mobility, invasiveness and differentiation (5). The expression of TIMP-1 was higher in malignant and borderline tumors than in benign tumors and strong TIMP-2 immunostaining has been found in serous ovarian carcinomas (6,7).

About 15 studies have been published on the prognostic value of MMP-2, MMP-7, MMP-9, MT1-MMP, TIMP-1 or TIMP-2 in ovarian cancers, but the results remain controversial. Most of these MMPs and TIMPs have been shown to be overexpressed in tumors, peritoneal implants or metastatic lesions, and associated with poor outcome (2,8-13). On the other hand, a strong MMP-2, MMP-9 or MMP-7 signal in cancer cells has been found to predict better survival (14–16). However, most of the previous studies evaluated MMPs and TIMPs separately in ovarian neoplasms, and little is known of their concomitant expression in epithelial ovarian cancers. We therefore, used a translational approach to analyze the epithelial and stromal expressions of MMP-2, MMP-7, MMP-9, MT1-MMP, TIMP-1 and TIMP-2 in advanced epithelial ovarian cancers and to assess their prognostic value.

## Materials and methods

### Patients and tumors

Ovarian tissue samples were obtained from all the patients who underwent surgery consecutively for FIGO stage III and IV epithelial ovarian cancer in the Gynecology Department of Tenon Hospital, Paris, from 2001 to 2006.

All the tumors were reviewed to confirm histological diagnosis. Histological typing followed the FIGO recommendations (17). Epidemiological characteristics, recurrence and survival were recorded for all patients. The study was approved by the Ethics Committee of the College National des Gynécologues et Obstétriciens Français.

### Tissue microarray (TMA) and immunohistochemistry

Formalin-fixed, paraffin-embedded tumor samples were used to construct a TMA, as previously described (18). Briefly, after selection of a representative tumor region from each tumor block, tissue cylinders were punched with the use of a custom-made precision instrument (Beecher Instruments, Silver Spring, MD) and transferred to a 25 × 35 mm paraffin block under microscopic control. TMA blocks were cut into 4 μm sections and transferred to glass slides (19). Separate sections from the TMA blocks were used for immunohistochemical analysis, using the Ventana Nexes automated immunohistochemistry system (Ventana Medical Systems, Tucson, AZ).

Purified mouse monoclonal or rabbit polyclonal antibodies against human MMP-2, -7, -9, MT1-MMP, TIMP-1, and -2 were used as primary antibodies at various concentrations: MMP-2 (mouse; clone 42-5D11; Calbiochem, San Diego, CA; 5 μg/ml), MMP-7 (mouse; clone ID2; Lab Vision Corp., Fremont, CA; 1.3 μg/ml), MMP-9 (mouse; clone 56-2A4; Calbiochem; 20 μg/ml), MT1-MMP (rabbit; Lab Vision Corp.; 8 μg/ml), TIMP-1 (mouse; clone 102D1; Lab Vision Corp.; 8 μg/ml), and TIMP-2 (mouse; clone 3A4; Lab Vision Corp.; 4 μg/ml).

Prior to the primary antibody staining an antigen retrieval step was used combined with a high temperature antigen-unmasking technique (Dako Target Retrieval Solution, Glostrup, Denmark; 100°C, 30 min). For MMP-7, antigen unmasking was achieved with proteinase K, 4 min. The automated procedure is based on an indirect biotin-avidin system with a universal biotinylated immunoglobulin as secondary antibody, diaminobenzidine as substrate, and hematoxylin as counterstain. Except for MT1-MMP, a Ventana amplification kit was used in addition to the automated procedure (Ventana Medical Systems).

Positive controls for MMP-2, MMP-7, MMP-9, MT1-MMP, TIMP-1 and TIMP-2 were sections of endometrial cancers which had been strongly stained in a previous study (20). For negative control, the primary antibody was replaced by an irrelevant non-immune mouse antibody of the same immunoglobulin G subtype.

### Semiquantitative analysis

The TMA was analyzed by light microscopy by use of a ×10 objective. Immunostaining results were scored by JLB and AC independently, using the HSCORE (21). The HSCORE was produced by multiplying the percentage of stained tumor cells (0–100%) with the intensity score. The intensity of staining was scored on a 4-point scale: 0, no staining; 1, weak; 2, moderate; 3, intense. Thus, each score ranged 0–300. For each tumor specimen, the HSCORE of a given MMP or TIMP was assessed in epithelial and stromal cells. Discordance between the two examiners never exceeded 5%.

### Statistical analysis

Continuous variables were compared with Student’s t-test and categorical variables were compared with the χ^2^ test or Fisher’s exact test, as appropriate. P-values <0.05 were considered statistically significant. Because of multiple comparisons in MMP and TIMP expressions, Bonferroni correction was used to determine the significance levels of two-tailed P-values (22). This was achieved by dividing the common P-value border 0.05 by the number of comparisons.

Unsupervised analysis was performed to organize the results of TMA immunostaining into meaningful structures. Classical Multidimensional Scaling (CMDS), also known as Principal Component Analysis, is a transformation procedure used to reduce multidimensional data sets to lower dimensions for analysis. This procedure provides information about the spatial ‘proximity’ (similarity) or ‘distance’ (difference) in a multidimensional space where each dimension is one marker of the panel. Results of CMDS can be plotted into the most informative two-dimensional construction in which one point represents one tumor.

The performance of each MMP was quantified with respect to discrimination. Discrimination (i.e., whether the relative ranking of individual predictions is in the correct order) was quantified with the concordance index (C index) and its 95% confidence interval, which is similar to the area under the receiver operating characteristic curve (AUROC) but appropriate for censored data. The C index is the probability that given two randomly selected patients, the patient with the worse score will actually have a worse outcome. It ranges from 0 to 1, with 1 indicating perfect concordance, 0.5 indicating no better concordance than chance, and 0 indicating perfect discordance. The optimal threshold for each MMP was determined using the function ‘optimal.thresholds’ (PresenceAbsence) of the R package.

Survival curves were constructed according to Kaplan-Meier method. Survival was defined from primary surgery or the beginning of neoadjuvant chemotherapy to date of death and evaluated in 2007. Kaplan-Meier survival curves were calculated using univariate analysis and compared by the log-rank test. Statistical significance was set at P<0.05.

All analyses were performed using the R package (http://lib.stat.cmu.edu/R/CRAN/).

## Results

### Patients and tumors

Out of the 69 patients included, 43 (62%) underwent primary surgery and the remaining 26 (38%) were treated by neoadjuvant chemotherapy followed by interval surgery. Clinical and biological characteristics were not significantly different between the groups: median age 64 years (range 26–84) and 62 years (range 21–76) respectively; FIGO stages III in 41/43 patients (95%) and 22/26 patients (85%), respectively; peritoneal carcinomatosis in 35/43 patients (81%) and 18/26 patients (69%), respectively; median CA125 plasmatic level 395 U/ml (range 43–8845) and 988 U/ml (range 34–31210), respectively.

**Figure 1:**
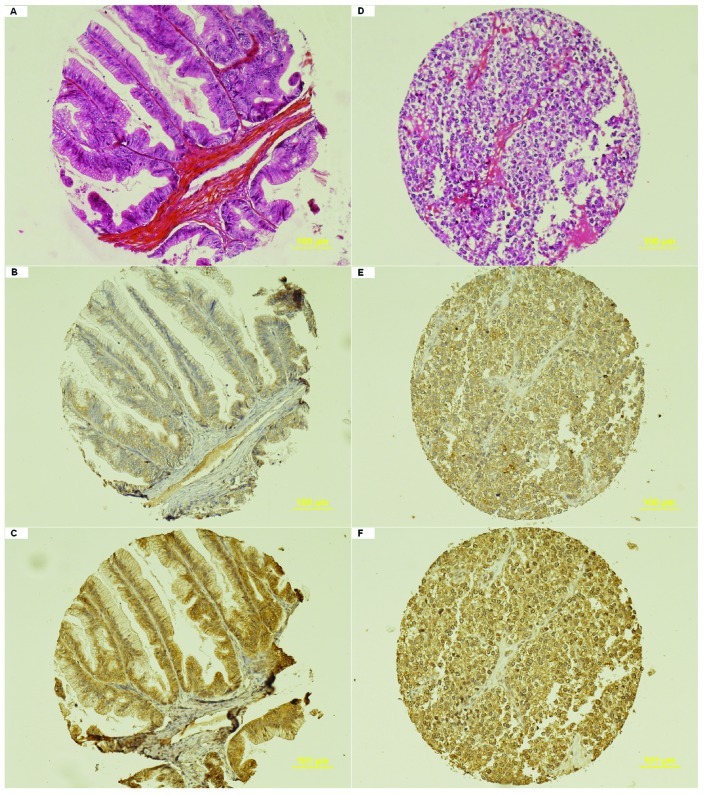
Immunohistochemical analysis of two specimens of the tissue microarray. Representative cases showing haematoxylin-eosin stained sections (A and D), MMP-9 expression (B and E) and TIMP-2 expression (C and F). The specimen in the left column is a mucinous grade 1 carcinoma treated by primary surgery with optimal cytoreduction; epithelial MMP-9 (B) and TIMP-2 (C) expressions are 70 and 180, respectively. The specimen in the right column is a clear cell grade 3 carcinoma treated by primary surgery with suboptimal cytoreduction; epithelial MMP-9 (E) and TIMP-2 (F) expressions are 190 and 200, respectively.

Epithelial and stromal expressions of MMPs and TIMPs were determined in 92% (range, 89–94) and 98% (range, 97–99) of tissue samples. Immunostaining data for all markers were available in 58 of the 69 patients (84%). The mean (SD) epithelial HSCORE values of MMPs and TIMPs were as follows: MMP-2, 96 (47); MMP-7, 66 (59); MMP-9, 133 (47); MT1-MMP, 120 (67); TIMP-1, 84 (44); TIMP-2, 151 (46). The mean (SD) stromal HSCORE values of MMPs and TIMPs were as follows: MMP-2, 31 (40); MMP-7, 11 (19); MMP-9, 25 (19); MT1-MMP, 11 (12); TIMP-1, 6 (8); TIMP-2, 31 (23). Representative cases of haematoxylin-eosin stained sections, MMP-9 expression and TIMP-2 expression in two specimens are shown in Fig. 1.

The histological and immunohistological characteristics of the tumors according to primary or interval surgery are shown in Table I. No histological difference was observed between the groups except for tumor size which was significantly reduced after neoadjuvant chemotherapy. Immunohistochemistry showed epithelial expressions of MMPs and TIMPs to be higher in tumors from patients treated by primary surgery than those undergoing interval surgery. P-values <0.05 were obtained for epithelial MT1-MMP and TIMP-2. However, none of these differences remained significant after Bonferroni correction.

### Cytoreductive surgery

Cytoreductive surgery was considered optimal (residual tumor ≤1 cm) in 27/43 patients (63%) treated by primary surgery and in 18/26 patients (69%) treated by interval surgery.

Clinical and biological characteristics (age, FIGO stage, CA125 plasmatic level) were not significantly different in patients with optimal cytoreduction and in patients with suboptimal cytoreduction. However, peritoneal carcinomatosis was present in 30/45 patients (66%) with optimal cytoreduction and in 22/24 patients (96%) with suboptimal cytoreduction (P=0.007).

The histological and immunohistological characteristics of the tumors according to optimal or suboptimal cytoreduction are shown in Table II. There was no significant histological difference between the groups. Lymphovascular space involvement tended to be more present in tumors from patients with suboptimal cytoreduction. Immunohistochemistry showed that expressions of epithelial MMP-9 and TIMP-2 were higher in patients with suboptimal cytoreduction than in patients with optimal cytoreduction (P<0.05). However, only the difference in epithelial MMP-9 was considered significant after Bonferroni correction. The epithelial and stromal levels of the other MMPs and TIMPs were not different in tumors from patients with optimal or suboptimal cytoreduction.

In patients treated by primary surgery, the expression of epithelial MMP-9 was higher in patients with suboptimal cytoreduction than in patients with optimal cytoreduction: mean (SD) HSCORE values 160 (45) and 127 (41), respectively, P=0.019. In patients treated by interval surgery, the epithelial and stromal levels of MMPs and TIMPs were not different in tumors from patients with optimal or suboptimal cytoreduction.

**Figure 2:**
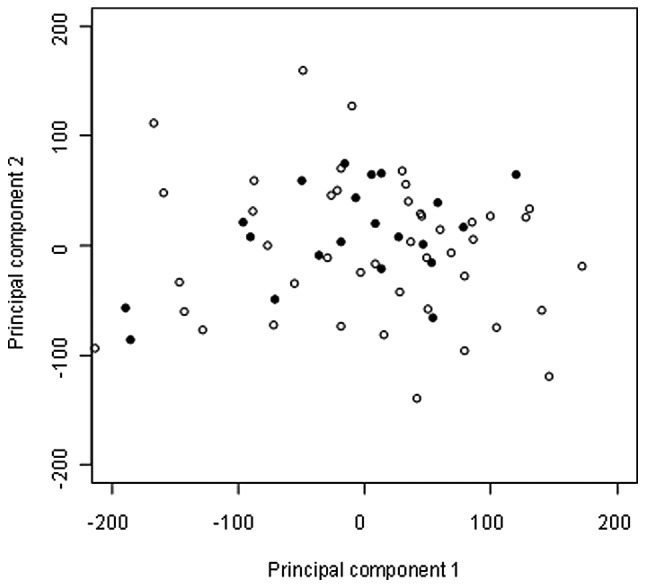
Two dimensional representation of ovarian tumors by classical multidimensional scaling (principal component analysis). Patient tumors with optimal cytoreduction are represented by circles and patient tumors with suboptimal cytoreduction are plotted in black.

CMDS showed that the tumors distributed homogeneously, independently from the surgical outcome (Fig. 2) indicating that the outcome of cytoreductive surgery was poorly influenced by MMP and TIMP profile of the tumors.

### Survival

Survival was assessed according to clinical, histological and immunohistological data. The median follow-up was 24 months (range 7–78). As expected, the 2-year survival probability differed significantly according to surgical outcome: 86% in patients with optimal cytoreduction and 63% in patients with suboptimal cytoreduction (Table III). Survival was also negatively influenced by tumors with clear cell/other histological types or with lymph node involvement. However, lymphovascular space involvement did not influence survival.

The performance of MMPs and TIMPs for survival quantified with the C index was poor. The AUROC values ranged from 0.48 to 0.65 (Table IV). P-values <0.05 were obtained for epithelial MT1-MMP and TIMP-2. However, none of these differences remained significant after Bonferroni correction. Survival was negatively influenced by positive expressions of epithelial TIMP-2 and stromal TIMP-1. Again, none of these differences remained significant after Bonferroni correction.

## Discussion

This study confirms the relevance of MMPs and TIMPs as markers of tumor proliferation and invasiveness as their epithelial expressions were relatively high in advanced ovarian cancers. However, we failed to demonstrate that they were of any prognostic value as the distribution of the tumors was scattered in the CMDS model and not influenced by the quality of cytoreduction. In addition, MMP and TIMP expressions were found not to influence survival.

In the present study, MMP and TIMP expressions were higher in the epithelium than in the stroma of ovarian cancers with mean HSCORE values ranging from 66–151 and 6–31, respectively. It has already been demonstrated that high levels of epithelial MMPs and TIMPs are not actually specific for malignant tumors. For example, MMP-2 signal was shown to be present in 76% of malignant tumors and 54% of benign tumors on immunohistochemical analysis (2). Another study found MMP-2 to be more frequently expressed in benign tumors with morphological altered lesions than in established carcinomas (23). Similar MMP-7 profiles have been observed in mucinous tumors whatever their benign, borderline or malignant nature (24). MT1-MMP mRNA was shown to be strongly expressed both in borderline and malignant tumors (25). High TIMP-2 expressions have been found in malignant tumors compared to borderline and benign ovarian tumors while various expressions were observed for TIMP-1 in the same figure (26,27). MMP-9 is probably the least controversial. Stronger epithelial MMP-9 expressions have been reported in malignant tumors than in benign or borderline tumors (28,29).

These discrepancies between studies can be partly explained by different methods of analysis and detection and also because the MMP and TIMP values were evaluated separately rather than by a translational approach. In a previous study using the same scoring system, we found alterations in all MMP and TIMP expressions in malignant serous tumors compared to benign and borderline serous tumors in univariate analysis though the alterations were less marked in malignant mucinous tumors (18). By cluster analysis, only MT1-MMP, MMP-7 and MMP-9 could distinguish malignant serous tumors from borderline and benign ovarian tumors. By CMDS analysis, the tumors were distributed first according to the mucinous or serous histological type, then according to the benign, borderline or malignant nature of the tumor. The influence of histological type has also been underlined in a study showing high level expressions of MMP-2 and MT1-MMP in ovarian clear cell carcinoma relative to other histotypes (30).

In the present study, tumors from patients with optimal cytoreduction demonstrated low epithelial MMP-9 expression by univariate analysis. The other expressions were not discriminative. In addition, CMDS failed to demonstrate any influence of MMPs and TIMPs on the quality of cytoreduction.

The prognostic impact of MMP-9 has been underlined in two studies assessing the latent and the active forms of gelatinases by zymography. In one study, it was found that only MMP-9, but not MMP-2, had an impact on recurrence (31). Activities of proMMP-9 (the latent form of MMP-9) and active MMP-9 in the tumor were higher in patients with recurrence. However, no values were available on the quality of cytoreduction in this study. In the other study, only proMMP-9 had an impact on prognosis (32). ProMMP-9 was associated with short survival only in the subgroup of patients with optimal cytoreduction, while for patients with residual disease proMMP-9 did not predict survival. Therefore, proMMP-9 is of strong prognostic power in patients with no postoperative residual disease.

By immunohistochemistry, epithelial MMP-9 has been associated with no or ≤2 cm residual disease only if there is a strong signal in a high proportion of cancer cells (16). In addition, low stromal MMP-9 expression was significantly related to tumors with no residual disease. These results contrast with ours and the literature. However, we demonstrate in the present study that MMP-9 loses its prognostic impact in terms of cytoreduction once a translation approach is adopted.

MMP and TIMP expressions did not influence survival in our study population. Their prognostic values were outweighed by clinical and histological data such as tumor type, lymph node involvement and cytoreduction. Our study is the first to assess the concomitant expressions of 6 MMPs and TIMPs in the prognosis of ovarian cancer. Most of the other trials evaluating prognosis not only analyzed MMPs and TIMPs selectively but also only investigated 1 to 3 MMPs or TIMPs. Studies including more than 4 MMPs or TIMPs have mainly dealt with physiopathology.

One study questioned the association between the expression of 4 MMPs or TIMPs and disease outcome (8–10). By univariate analysis, high levels of MMP-2, MMP-9, MT1-MMP and TIMP-2, were detected by immunohistochemistry and/or mRNA *in situ* hybridization in tumor cells from peritoneal or pleural effusions, metastatic lesions and primary ovarian tumors. A multivariate analysis was carried out in one of the reports on primary tumors (8). Surprisingly, epithelial MMP-9 and stromal TIMP-2 expressions correlated with poor survival, while tumor type, grade and stage did not. In another study assessing MMP-2, MMP-9 and MT1-MMP in 77 patients with ovarian cancer, strong epithelial MT1-MMP and stromal MMP-9 and FIGO stage were independently associated with shorter survival (33). On the other hand, as in our study, clinical (FIGO stage, residual disease) and histological (grade) characteristics were found to be independent prognostic factors and outweighed MMP-9 epithelial and stromal expressions which influenced clinical outcome in univariate analysis (16).

Discrepancies may be explained by the absence of a given threshold for each MMP evaluated by immunohistochemistry as this differs from study to study (16,33). We used the concordance index to evaluate the discrimination of quantitative values of MMPs and TIMPs for survival. With this approach, the alternatives are compared directly, without having to set a possibly arbitrary threshold. In addition, none of the studies mentioned above referred to the Bonferroni correction of P-values in case of multiple tests. Thus, the significance of certain MMPs or TIMPs could be overestimated.

Technically, immunohistochemistry procedures require multiple steps which may cause concern. In a recent study, the HSCORE technique was used to assess the prognostic significance of six biomarkers including gelatinases in a TMA of 185 specimens (34). Unfortunately, MMP-2 and MMP-9 staining was observed at very low levels in serous and endometrioid carcinomas; thus, gelatinase expressions failed to demonstrate any influence in contrast to the other biomarkers studied. In our center, numerous tests were run before finding the best method for antigen unmasking, to select the concentration of primary antibody staining, and to test the advantage of using the Ventana amplification kit.

To conclude, standard statistical analysis adjusted after Bonferroni correction as well as classical multidimensional scaling failed to demonstrate any influence of MMPs and TIMPs in the prognosis of ovarian tumors. Despite the lack of standardized methods for MMP and TIMP detection and the underuse of translational approach in the literature, the impact of MMPs and TIMPs on prognosis is probably less important than previously thought. Further studies must address these concerns as considerable hopes are pinned on the success of targeted therapy in patients with advanced ovarian cancer.

## Figures and Tables

**Table I:** Histological and immunohistological characteristics of the tumors according to primary or interval surgery.

Tumor characteristics	Primary surgery (n=43) (%)	Interval surgery (n=26) (%)	P-value^a^
Tumor size (mm)	96 (47)	69 (53)	**0.042**
Histology			
Serous (n=40)	24 (56)	16 (61)	0.236
Mucinous (n=2)	1 (2)	1 (4)	
Endometrioid (n=15)	9 (21)	6 (23)	
Clear cells (n=7)	7 (16)	0	
Other (n=5)	2 (5)	3 (12)	
Grade			
Well differentiated (n=7)	5 (12)	2 (8)	0.867
Moderately differentiated (n=32)	19 (44)	13 (50)	
Poorly differentiated (n=26)	17 (39)	9 (34)	
Not determined (n=4)	2 (5)	2 (8)	
Lymphovascular space involvement			
No (n=46)	26 (61)	20 (77)	0.320
Yes (n=22)	16 (37)	6 (23)	
Not determined (n=1)	1 (2)	0	
Lymph node involvement when sampled			
Not involved, N^−^ (n=15)	8 (33)	7 (47)	0.405
Involved, N^+^ (n=24)	16 (66)	8 (53)	
MMP-2			
Epithelial	98 (43)	93 (54)	0.689
Stromal	25 (37)	41 (43)	0.142
MMP-7			
Epithelial	73 (66)	54 (43)	0.192
Stromal	9 (16)	15 (23)	0.217
MMP-9			
Epithelial	139 (45)	121 (48)	0.125
Stromal	25 (21)	25 (17)	0.878
MT1-MMP			
Epithelial	132 (72)	97 (49)	0.049
Stromal	12 (15)	8 (7)	0.232
TIMP-1			
Epithelial	85 (46)	82 (42)	0.804
Stromal	6 (8)	7 (8)	0.609
TIMP-2			
Epithelial	159 (44)	135 (47)	0.041
Stromal	31 (26)	31 (17)	0.990

Quantitative values are reported by using mean (SD).

a P-value <0.05 was considered as significant for histological data. P-value <0.004 was considered as significant for immunohistological data after Bonferroni correction. Bold text, statistically significant.

**Table II:** Histological and immunohistological characteristics of the tumors according to optimal or suboptimal cytoreduction.

Characteristics	Optimal cytoreduction (n=45) (%)	Suboptimal cytoreduction (n=24) (%)	P-value^a^
Mean tumor size, mm (SD)	86 (51)	87 (51)	0.907
	75 (25–220)	65 (25–180)	0.839
Histology			
Serous (n=41)	27 (60)	13 (54)	0.885
Mucinous (n=2)	1 (2)	1 (4)	
Endometrioid (n=15)	9 (20)	6 (25)	
Clear cells (n=7)	4 (9)	3 (13)	
Other (n=5)	4 (9)	1 (4)	
Grade			
Well differentiated (n=7)	5 (11)	2 (8)	0.629
Moderately differentiated (n=32)	22 (51)	10 (42)	
Poorly differentiated (n=26)	15 (38)	11 (50)	
Lymphovascular space involvement			
No (n=46)	34 (76)	12 (52)	0.051
Yes (n=22)	11 (24)	11 (48)	
Lymph node involvement when sampled			
Not involved, N^−^ (n=15)	14 (40)	1 (25)	1.000
Involved, N^+^ (n=24)	21 (60)	3 (75)	
MMP-2			
Epithelial	94 (52)	100 (37)	0.608
Stromal	25 (27)	42 (55)	0.082
MMP-7			
Epithelial	67 (50)	65 (74)	0.889
Stromal	13 (20)	8 (16)	0.268
MMP-9			
Epithelial	120 (43)	156 (45)	**0.002**
Stromal	22 (20)	30 (17)	0.120
MT1-MMP			
Epithelial	115 (71)	130 (58)	0.398
Stromal	11 (14)	10 (9)	0.749
TIMP-1			
Epithelial	84 (49)	84 (33)	0.974
Stromal	6 (8)	6 (8)	0.932
TIMP-2			
Epithelial	142 (48)	168 (38)	0.026
Stromal	30 (25)	32 (19)	0.682

Quantitative values are reported by using mean (SD).

a P-value <0.05 was considered as significant for histological data. P-value <0.004 was considered as significant for immunohistological data after Bonferroni correction. Bold text, statistically significant.

**Table III:** Patient and tumor characteristics and survival outcome.

Characteristics	Disease-free 2-year survival (%)	P-value^a^ log-rank
Histology		**0.004**
Serous (n=41)	80	
Mucinous (n=2)	NA	
Endometrioid (n=15)	87	
Clear cells (n=7)	67	
Other (n=3)	33	
Grade		0.874
Well differentiated (n=7)	100	
Moderately differentiated (n=32)	75	
Poorly differentiated (n=29)	73	
Lymphovascular space involvement		0.963
No (n=76)	78	
Yes (n=22)	74	
Lymph node		**<0.001**
Not involved, N^−^ (n=15)	100	
Involved, N^+^ (n=24)	81	
Not sampled, Nx (n=30)	61	
FIGO stage		0.492
III (n=63)	76	
IV (n=6)	83	
Cytoreduction		**0.002**
Optimal (n=45)	86	
Suboptimal (n=24)	63	

a P-value <0.05 was considered as significant for clinical and histological data. Bold text, statistically significant.

**Table IV:** MMP and TIMP expressions in tumors and survival outcome.

	Concordance index^a^		2-year disease-free survival (%)		
		
Characteristics	AUC	P-value	Negative expression^b^	Positive expression^c^	P-value^d^ log-rank
MMP-2					
Epithelial	0.51	0.351	53.8	57.8	0.331
Stromal	0.57	0.072	54.4	54.8	0.472
MMP-7					
Epithelial	0.51	0.473	54.8	46.2	0.864
Stromal	0.48	0.590	52.5	58.3	0.988
MMP-9					
Epithelial	0.51	0.394	53.7	54.9	0.902
Stromal	0.56	0.077	58.6	51.4	0.302
MT1-MMP					
Epithelial	0.65	0.007	56.2	48	0.371
Stromal	0.51	0.390	52.0	56.1	0.402
TIMP-1					
Epithelial	0.51	0.491	51.0	74	0.093
Stromal	0.57	0.078	58.1	40.2	0.016
TIMP-2					
Epithelial	0.61	0.023	72.2	47.9	0.050
Stromal	0.57	0.077	54.4	59.0	0.456

a Discrimination of the performance of each MMPs and TIMPs for survival was quantified with the concordance index (C index), which is similar to the area under the receiver operating characteristic curve (AUROC). Different values of the AUROC are close to 0.5, indicating no better concordance than chance.

b Negative expression was defined by a value under the optimal threshold.

c Positive expression was defined by a value over the optimal threshold.

d P-value <0.004 was considered as significant for immunohistological data after Bonferroni correction. No difference in 2-year disease-free survival was observed for MMP and TIMP expressions after Bonferroni correction.

## References

[1] Schröpfer A, Kammerer U, Kapp M, Dietl J, Feix S, Anacker J (2010). Expression pattern of matrix metalloproteinases in human gynecological cancer cell lines. BMC Cancer.

[2] Wu X, Li H, Kang L, Wang W, Shan B (2002). Activated matrix metalloproteinase-2: a potential marker of prognosis for epithelial ovarian cancer. Gynecol Oncol.

[3] Planagumà J, Liljeström M, Alameda F (2011). Matrix metalloproteinase-2 and matrix metalloproteinase-9 codistribute with transcription factors RUNX1/AML1 and ETV5/ERM at the invasive front of endometrial and ovarian carcinoma. Hum Pathol.

[4] Wang FQ, So J, Reierstad S, Fishman DA (2005). Matrilysin (MMP-7) promotes invasion of ovarian cancer cells by activation of progelatinase. Int J Cancer.

[5] Moss NM, Barbolina MV, Liu Y, Sun L, Munshi HG, Stack MS (2009). Ovarian cancer cell detachment and multicellular aggregate formation are regulated by membrane type 1 matrix metalloproteinase: a potential role in I.p. metastatic dissemination. Cancer Res.

[6] Huang LW, Garrett AP, Bell DA, Welch WR, Berkowitz RS, Mok SC (2000). Differential expression of matrix metalloproteinase-9 and tissue inhibitor of metalloproteinase-1 protein and mRNA in epithelial ovarian tumors. Gynecol Oncol.

[7] Kim TJ, Rho SB, Choi YL (2006). High expression of tissue inhibitor of metalloproteinase-2 in serous ovarian carcinomas and the role of this expression in ovarian tumorigenesis. Hum Pathol.

[8] Davidson B, Goldberg I, Gotlieb WH (1999). High levels of MMP-2, MMP-9, MT1-MMP and TIMP-2 mRNA correlate with poor survival in ovarian carcinoma. Clin Exp Metastasis.

[9] Davidson B, Reich R, Berner A (2001). Ovarian carcinoma cells in serous effusions show altered MMP-2 and TIMP-2 mRNA levels. Eur J Cancer.

[10] Davidson B, Goldberg I, Gotlieb WH, Kopolovic J, Ben-Baruch G, Nesland JM, Reich R (2002). The prognostic value of metalloproteinases and angiogenic factors in ovarian carcinoma. Mol Cell Endocrinol.

[11] Kamat AA, Fletcher M, Gruman LM (2006). The clinical relevance of stromal matrix metalloproteinase expression in ovarian cancer. Clin Cancer Res.

[12] Périgny M, Bairati I, Harvey I (2008). Role of immunohistochemical overexpression of matrix metalloproteinases MMP-2 and MMP-11 in the prognosis of death by ovarian cancer. Am J Clin Pathol.

[13] Rauvala M, Puistola U, Turpeenniemi-Hujanen T (2005). Gelatinases and their tissue inhibitors in ovarian tumors; TIMP-1 is a predictive as well as a prognostic factor. Gynecol Oncol.

[14] Sillanpää SM, Anttila MA, Voutilainen KA (2006). Prognostic significance of matrix metalloproteinase-7 in epithelial ovarian cancer and its relation to β-catenin. Int J Cancer.

[15] Sillanpää S, Anttila M, Suhonen K (2007). Prognostic significance of extracellular matrix metalloproteinase inducer and matrix metalloproteinase 2 in epithelial ovarian cancer. Tumour Biol.

[16] Sillanpää S, Anttila M, Voutilainen K (2007). Prognostic significance of matrix metalloproteinase-9 (MMP-9) in epithelial ovarian cancer. Gynecol Oncol.

[17] Benedet JL, Bender H, Jones H, Ngan HYS, Pecorelli S (2000). FIGO staging classifications and clinical practice guidelines in the management of gynecologic cancers. FIGO Committee on Gynecologic Oncology. Int J Gynecol Obstet.

[18] Brun JL, Cortez A, Commo F, Uzan S, Rouzier R, Daraï E (2008). Serous and mucinous ovarian tumors express different profiles of MMP-2, -7, -9, MT1-MMP, and TIMP-1 and -2. Int J Oncol.

[19] Skacel M, Skilton B, Pettay JD, Tubbs RR (2002). Tissue microarrays: a powerful tool for high-throughput analysis of clinical specimen: a review of the method with validation data. Appl Immunohistochem Mol Morphol.

[20] Graesslin O, Cortez A, Fauvet R, Lorenzato M, Birembaut P, Darai E (2006). Metalloproteinase-2, -7 and -9 and tissue inhibitor of metalloproteinase-1 and -2 expressions in normal, hyperplastic and neoplastic endometrium: a clinical-pathological correlation study. Ann Oncol.

[21] Budwit-Novotny DA, McCarty KS, Cox EB (1986). Immunohistochemical analyses of estrogen receptor in endometrial adenocarcinoma using a monoclonal antibody. Cancer Res.

[22] Bland JM, Altman DG (1995). Multiple significance tests: the Bonferroni method. BMJ.

[23] Cai KQ, Yang WL, Capo-Chichi CD (2007). Prominent expression of metalloproteinases in early stages of ovarian tumorigenesis. Mol Carcinogen.

[24] Shigemasa K, Tanimoto H, Sakata K (2000). Induction of matrix metalloprotease-7 is common in mucinous ovarian tumors including early stage disease. Med Oncol.

[25] Afzal S, Lalani EN, Poulsom R (1998). MT1-MMP and MMP-2 mRNA expression in human ovarian tumors: possible implications for the role of desmoplastic fibroblasts. Hum Pathol.

[26] Sakata K, Shigemasa K, Nagai N, Ohama K (2000). Expression of matrix metalloproteinases (MMP-2, MMP-9, MT1-MMP) and their inhibitors (TIMP-1, TIMP-2) in common epithelial tumors of the ovary. Int J Oncol.

[27] Määttä M, Santala M, Soini Y, Talvensaari-Mattila A, Turpeenniemi-Hujanen T (2004). Matrix metalloproteinases 2 and 9 and their tissue inhibitors in low malignant potential ovarian tumors. Tumor Biol.

[28] Behrens P, Rothe M, Florin A, Wellman A, Wernert N (2001). Invasive properties of serous human epithelial ovarian tumors are related to Ets-1, MMP-1 and MMP-9 expression. Int J Mol Med.

[29] Ozalp S, Tanir HM, Yalcin OT, Kabukcuoglu S, Oner U, Uray M (2003). Prognostic value of matrix metalloproteinase-9 (gelatinase B) expression in epithelial ovarian tumors. Eur J Gynaecol Oncol.

[30] Adley BP, Gleason KJ, Yang XJ, Stack MS (2009). Expression of membrane type 1 matrix metalloproteinase (MMP-14) in epithelial ovarian cancer: high level expression in clear cell carcinoma. Gynecol Oncol.

[31] Demeter A, Sziller I, Csapo Z (2005). Molecular prognostic markers in recurrent and in non-recurrent epithelial ovarian cancer. Anticancer Res.

[32] Lengyel E, Schmalfeldt B, Konik E (2001). Expression of latent matrix metalloproteinase 9 (MMP-9) predicts survival in advanced ovarian cancer. Gynecol Oncol.

[33] Lin YG, Han LY, Kamat AA (2007). EphA2 overexpression is associated with angiogenesis in ovarian cancer. Cancer.

[34] Lin CK, Chao TK, Yu CP, Yu MH, Jin JS (2009). The expression of six biomarkers in the four most common ovarian cancers: correlation with clinicopathological parameters. APMIS.

